# Valorization of Spent Millet Flour in Composite Bread: Characterization, Predictive Modeling, and Multi‐Objective Optimization for Nutritional Enhancement

**DOI:** 10.1002/fsn3.71843

**Published:** 2026-04-29

**Authors:** Afia Sakyiwaa Amponsah, Gladys Kyere, Kwadwo Boateng Prempeh, Kwadwo Adinkrah‐Appiah

**Affiliations:** ^1^ Faculty of Applied Science and Technology Sunyani Technical University Sunyani Ghana; ^2^ Department of Food and Nutrition Education University of Education Winneba Ghana; ^3^ Faculty of Business and Management Studies Sunyani Technical University Sunyani Ghana; ^4^ Faculty of Engineering Sunyani Technical University Sunyani Ghana

**Keywords:** composite bread, desirability function, food waste valorization, multi‐objective optimization, multivariate analysis, predictive modeling, response surface methodology

## Abstract

This study valorized spent millet flour (SMF) from traditional pito beverage processing by incorporating it into composite bread formulations. SMF was prepared by oven‐drying the pito residue at 60°C, dry‐milling, and sieving through a 250 μm mesh to achieve a standardized particle size suitable for bread applications. Response surface methodology (RSM) was employed to develop predictive models and identify optimal substitution levels. SMF exhibited superior nutritional composition compared to wheat flour, with higher crude protein (12.8% vs. 11.5%), crude fiber (8.7% vs. 2.1%), and ash content (2.8% vs. 0.6%), alongside a 33% greater water absorption capacity attributed to fermentation‐induced protein modification and fiber concentration. Composite bread was prepared at 10%, 20%, and 30% SMF substitution levels and compared against a 100% wheat flour control bread. Second‐order polynomial models developed for twelve quality responses achieved high predictive accuracy (*R* ^2^ = 0.912–0.988) with non‐significant lack‐of‐fit tests (*p* > 0.05) and experimental validation errors below 4.5%. Multi‐objective desirability function optimization identified 14.2% substitution as globally optimal (*D* = 0.847), delivering a 9.8% protein increase and 68.4% fiber increase while maintaining consumer acceptability at 7.21/9.0. The reduction in loaf volume with increasing SMF substitution is attributable to a dual mechanism: (i) gluten dilution, whereby replacement of wheat protein with non‐gluten millet proteins reduces the viscoelastic network required for gas retention, and (ii) physical disruption of the gas‐cell wall structure by coarse insoluble bran particles in SMF, which puncture developing gas cells during proofing and early baking stages. Principal component analysis, hierarchical cluster analysis, and Pearson correlation confirmed the mechanistic trade‐off between nutritional enhancement and structural bread quality, with fiber content emerging as the dominant predictor of volume reduction (*r* = −0.89, *p* < 0.01). The RSM optimization framework demonstrated here is transferable to other fermented cereal by‐product systems across sub‐Saharan Africa.

## Graphical abstract

This study presents the circular valorization pathway from spent millet flour (SMF) generation during traditional pito beverage processing to its incorporation into composite wheat bread. The workflow is presented in four sequential stages: (1) By‐product recovery: SMF is collected from pito residues, oven‐dried at 60°C, milled, and sieved to 250 μm; (2) Flour characterization: SMF is compared against wheat flour for proximate composition (protein, fiber, ash) and functional properties (water absorption capacity, pasting viscosity); (3) RSM modeling: second‐order polynomial models are developed for 12 bread quality responses across 10% to 30% SMF substitution levels (*R* ^2^ = 0.912 to 0.988); and (4) Multi‐objective optimization: the Derringer‐Suich desirability function identifies 14.2% SMF substitution as globally optimal (*D* = 0.847), delivering a 9.8% increase in protein, a 68.4% increase in dietary fiber, and a consumer acceptability score of 7.21/9.0. Multivariate analysis (PCA, hierarchical clustering, Pearson correlation) confirms a mechanistic trade‐off between nutritional enrichment and structural bread quality, with fiber content as the dominant predictor of loaf volume reduction (*r* = −0.89). The framework supports circular economy principles by transforming a low‐value agro‐processing residue into a nutritionally enhanced staple food product.

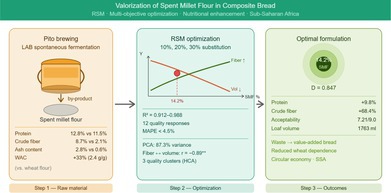

## 1 Introduction

Food waste is a global challenge, with one‐third of all food produced for human consumption left unconsumed. (Dey et al. 2024). In the cereal processing industry, substantial quantities of nutrient‐rich by‐products are generated during various operations, but are frequently underutilized, despite retaining considerable nutritional value (Fărcaș, Socaci, Nemeș, Salanță, et al. 2022; Fărcaș, Socaci, Nemeș, Pop, et al. 2022). An estimated 12.9% of all food waste is generated as a result of the processing of cereals, and 30% of the weight basis is also wasted (Fărcaș, Socaci, Nemeș, Salanță, et al. 2022; Fărcaș, Socaci, Nemeș, Pop, et al. 2022). This volume of agricultural waste from diverse cereal sources constitutes an environmental burden if improperly disposed of, yet also represents an untapped resource for value creation (Oyedeji et al. 2024).

Millet ( *Pennisetum glaucum* ) is of cultural and economic importance in Ghana's food system, serving as a key ingredient in traditional fermented beverages such as the indigenous beer ‘pito’ and non‐alcoholic malted drinks ‘Asaana’, which are widely consumed for nutritional, social, and ceremonial purposes (Madilo et al. 2024). The production of these traditional beverages generates substantial quantities of spent millet flour annually through a multistage process that includes grain malting (germination for 3–4 days), drying, milling, mashing with water, and filtration to extract fermentable liquids, leaving solid residues. These spent flours, representing approximately 20%–30% of the original grain weight, are the fiber‐rich solid residues remaining after extraction of soluble sugars and other water‐soluble components (Mussatto et al. 2006). Currently, spent millet flour is predominantly discarded or used for low‐value applications, such as animal feed, despite retaining considerable nutritional value (Czubaszek et al. 2021). Compositional analyses reveal that spent cereal flours contain elevated levels of protein (12%–18%), crude fiber (6%–12%), and ash (2%–4%) compared to their unprocessed counterparts, as the extraction process selectively removes soluble carbohydrates while concentrating the remaining nutrients (Fărcaș, Socaci, Nemeș, Salanță, et al. 2022; Fărcaș, Socaci, Nemeș, Pop, et al. 2022). Additionally, these flours retain bioactive compounds, including phenolic acids, flavonoids, and essential minerals such as iron, zinc, and magnesium, which contribute to their functional and nutritional potential (Gupta et al. 2025).

The challenge of agricultural waste accumulation presents both an environmental burden and an economic opportunity (Lackner and Besharati 2025). The valorization of spent cereal flours through incorporation into bread formulations aligns with circular economy principles by transforming waste materials into valuable resources while addressing multiple sustainability challenges simultaneously (Hafyan et al. 2024). Implementation of such a biorefinery platform for food waste is a promising approach, with the potential to reduce production costs relative to conventional wheat‐only formulations, enhance nutritional profiles, and reduce waste‐disposal requirements (Soares et al. 2024). Composite bread technology, involving partial substitution of wheat flour with alternative flours, has demonstrated significant potential to reduce import dependence while improving nutritional quality in various global contexts (Engindeniz and Bolatova 2021; Menon et al. 2015). However, the specific application of spent flours from traditional fermented beverage processing remains underexplored, particularly in West Africa, where these dual challenges of food waste and wheat dependency are most acute. From a nutritional perspective, millet possesses beneficial compositional characteristics, including higher fiber content, superior mineral profiles, and enhanced phytochemical diversity compared to wheat (Gupta et al. 2025). Even after beverage processing, where soluble carbohydrates are preferentially extracted during fermentation, spent flours retain substantial nutritional value through concentration effects (Gupta et al. 2025; Mussatto et al. 2006). This nutritional enhancement potential is particularly significant for addressing micronutrient deficiencies that remain prevalent in Ghana, where 66% of children under 5 years and 45% of reproductive‐age women suffer from iron deficiency anemia, and average dietary fiber intake (12–15 g/day) falls substantially below WHO recommendations of 25–30 g/day (Coomson and Aryeetey 2022).

The optimization of composite bread formulations incorporating agro‐industrial by‐products presents complex challenges requiring systematic mathematical approaches to balance competing quality and nutritional objectives. Traditional one‐factor‐at‐a‐time experimental approaches fail to capture non‐linear relationships and fail to identify true optima in multivariable food systems (Myers et al. 2016). Response surface methodology (RSM) addresses these limitations through systematic experimental design combined with regression modeling, enabling visualization of response surfaces, identification of optimal operating regions, and quantification of non‐linear relationships across multiple response variables simultaneously (Bezerra et al. 2008).

Existing RSM studies on composite bread have predominantly focused on single‐objective optimization, typically maximizing specific volume or sensory scores while treating nutritional enhancement as a secondary consideration (Sabanis and Tzia 2009). Spent millet flour from traditional fermented beverage processing is an understudied category of valorization substrate that differs fundamentally from brewer's spent grain (the primary comparator in international literature) due to differences in fermentation microbiota, processing duration, and substrate modification. Pito fermentation is primarily a spontaneous, lactic acid‐driven process dominated by heterofermentative lactic acid bacteria (LAB) such as Limosilactobacillus fermentum and Lactiplantibacillus plantarum, alongside *Saccharomyces cerevisiae* (Madilo et al. 2024). The organic acids and proteolytic enzymes produced by these microorganisms alter protein solubility and modify starch structure during mashing, resulting in a spent residue with reduced starch crystallinity and partially hydrolyzed protein fractions. These fermentation‐induced modifications improve the bioavailability of minerals previously bound in phytate complexes, and the concentration of soluble amino acids released during LAB proteolysis may enhance the nutritional quality of the residue beyond what crude protein analysis alone indicates. The technical challenge of incorporating spent millet flour into bread, however, is not merely nutritional. Millet is inherently gluten‐free, and spent millet flour is exceptionally high in insoluble dietary fiber. The absence of functional gluten proteins means that SMF cannot contribute to the viscoelastic dough network responsible for trapping carbon dioxide during yeast fermentation, while the insoluble fiber fraction physically disrupts existing gluten strands and competes with starch for available water. Together, these effects systematically impair gas retention and loaf volume with increasing substitution. This fundamental structural conflict between nutritional enrichment and baking performance is precisely why a complex, non‐linear RSM approach is necessary to identify the “sweet spot” where health gains and acceptable bread quality are simultaneously achievable. This study addresses three critical gaps. First, there is a lack of predictive mathematical models for spent millet flour–wheat bread systems. Second, the absence of multi‐objective optimization frameworks that simultaneously integrate nutrition and quality objectives, and third, the limited mechanistic understanding of how fermentation‐modified millet flour properties interact with bread quality formation.

The specific objectives of this study were to: (1) characterize the proximate composition and functional properties of spent millet flour and relate observed differences to fermentation‐induced structural modifications; (2) develop and validate second‐order polynomial models predicting twelve bread quality responses as functions of SMF substitution level; (3) identify the globally optimal substitution level through multi‐objective desirability function optimization integrating nutritional and sensory criteria; and (4) apply principal component analysis, hierarchical cluster analysis, and Pearson correlation to elucidate mechanistic relationships between flour properties and bread quality outcomes.

## 2 Materials and Methods

### 2.1 Sample Collection and Preparation

Spent millet flour was obtained from traditional beverage producers in Sunyani Municipality, Ghana. Fresh spent materials were collected within 24 h of beverage production, manually sorted to remove impurities, washed thoroughly with clean water (1:3 ratio), and drained. Materials were dried in a cabinet dryer (Gallenkamp OV‐440, UK) at 60°C ± 2°C for 18–24 h to achieve moisture content below 12%. Dried materials were milled using a laboratory hammer mill (Christy & Norris 8″ Lab Mill, UK) fitted with a 0.8 mm screen and sieved through 250 μm mesh. Commercial wheat flour, which served as the control and base for composite formulations and as the base for all other ingredients for bread making, was purchased from the Main Market in Sunyani, Bono Region, Ghana. The commercial wheat flour conforms to the standard specification for low‐extraction wheat flour and typically passes through a 150 μm (100‐mesh) sieve, consistent with standard milling practice (ACCA International 2010). The larger particle size of SMF (250 μm) relative to wheat flour (~150 μm) is acknowledged as a potential contributor to textural differences in composite bread; however, the 250 μm threshold was selected to reflect practical milling constraints in artisanal pito‐producing settings, and this particle size is within the range employed in comparable spent grain bread studies (Ktenioudaki et al. 2015; Stojceska and Ainsworth 2008).

#### 2.1.1 Bread Preparation

Composite bread was prepared using the straight‐dough method with modifications from Shongwe et al. (2022) where formulations were developed at four substitution levels: control (100% wheat flour), 10% spent millet flour substitution, 20% substitution, and 30% substitution. The base formulation, on a 100 g flour basis, consisted of a flour blend (100 g), instant dry yeast (2 g, *Saccharomyces cerevisiae*, SAF‐Instant), sugar (6 g), salt (1.5 g), margarine (3 g), and water adjusted to the flour's water absorption capacity. Since SMF exhibited a 33% higher water absorption capacity (2.4 g/g) relative to wheat flour (1.8 g/g), the actual water addition was adjusted proportionally for each substitution level to maintain equivalent dough hydration. The calculated water additions per 100 g flour blend were: 60 mL (0% SMF control), 62 mL (10% SMF), 64 mL (20% SMF), and 66 mL (30% SMF). These values were determined from the weighted average WAC of each flour blend and verified empirically through a preliminary consistency check using a farinograph (target dough development time ±0.5 min). Standardizing water addition to the blend's WAC ensured that observed differences in bread firmness, volume, and texture reflect the intrinsic structural effects of SMF incorporation rather than differential dough hydration levels.

**FIGURE 1:**
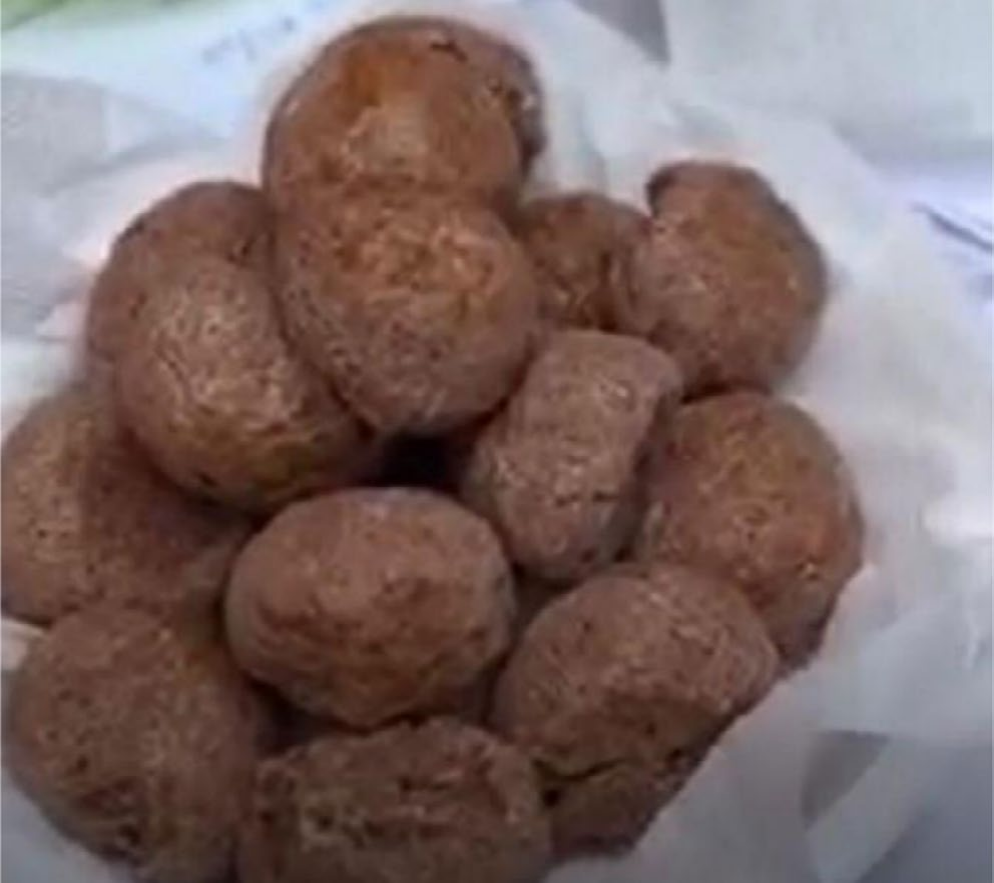
Millet‐wheat composite bread.

The mixing procedure involved: dry ingredients (flour, sugar, salt, yeast) were mixed in a planetary mixer (KitchenAid Professional 600, USA) using a dough hook at speed 2 for 2 min, margarine and water (temperature 28°C–30°C) were added gradually during mixing, and dough was developed at speed 4 for 8 min until smooth and elastic. Dough temperature was maintained at 28°C–30°C throughout mixing. The developed dough was transferred to lightly greased stainless steel bowls, covered with polyethene film, and fermented at 32°C for 60 min. After bulk fermentation, the dough was degassed by gentle pressing, divided into 300 g portions, rounded, and rested for 10 min. The dough pieces were shaped into cylindrical loaves, placed in greased bread pans (18 cm × 9 cm × 9 cm), and proofed under the same conditions for 45 min until doubled in volume. Baking was performed in a deck oven (MIWE Condo, Germany) at 200°C for 30 min. Baked loaves, as shown in Figure 1, were removed from pans immediately, cooled on wire racks for 2 h at room temperature, and stored in polyethene bags for subsequent analyses. Each formulation was prepared in triplicate.

### 2.2 Flour Characterization

#### 2.2.1 Proximate Composition Analysis

Proximate composition of flour samples was determined using Association of Official Analytical Chemists (AOAC) official methods conducted at the Biochemistry Laboratory, Kwame Nkrumah University of Science and Technology (KNUST), Kumasi, Ghana. All analyses were performed in triplicate, and results were expressed on a dry weight basis as percentages.

Moisture content was determined by the oven‐drying method (AOAC 930.15) as described by Ileleji et al. (2010) where approximately 5 g of ground sample was weighed into pre‐dried, pre‐weighed petri dishes and dried at 105°C for 4 h in a forced‐draft oven; samples were cooled in a desiccator and reweighed. Moisture content was calculated as percentage weight loss.

Crude protein determination used the Kjeldahl method (AOAC 2001.11) as described by Thiex (2009) where 1 g of samples was digested with concentrated sulfuric acid and catalyst tablets at 420°C for 2 h, the digests were diluted, distilled with 40% sodium hydroxide, and collected in boric acid solution. Titration against standardized 0.1 N hydrochloric acid determined nitrogen content, which was converted to crude protein using the factor 6.25.

Crude fat was extracted using the Soxhlet extraction method (AOAC 2003.05) (N. J. Thiex et al. 2003). Samples (5 g), wrapped in filter paper, were placed in Soxhlet extractors and extracted with petroleum ether for 6 h. Extraction flasks were dried at 105°C for 1 h, cooled in desiccators, and weighed to determine the fat content gravimetrically.

Total ash content was determined using the incineration method (AOAC 942.05) (Harris and Marshall 2017) samples (2 g) in preweighed porcelain crucibles were incinerated in a muffle furnace at 550°C for 2 h until white ash formed. Crucibles were cooled in desiccators and weighed to calculate the ash percentage.

Crude fiber was analyzed using the acid–base digestion method (AOAC 978.10) (Pathan et al. 2019); defatted samples (2 g) were digested sequentially with 1.25% sulfuric acid and 1.25% sodium hydroxide under reflux conditions (30 min each). Residues were washed, dried at 130°C, weighed, incinerated at 550°C, and reweighed. Fiber content was calculated as weight loss during incineration.

$$\begin{array}{c} \text{Carbohydrate content was determined}\;\mathrm{by}\;\text{difference}:\text{Carbohydrate}\;\left(\%\right)=\\ 100–\left(\%\text{moisture}+\%\text{protein}+\%\mathrm{fat}+\%\mathrm{ash}+\%\text{fiber}\right). \end{array}$$

### 2.3 Functional Properties

#### 2.3.1 Water Absorption Capacity (WAC)

Water absorption capacity (WAC) was determined using the method described by Kaur and Singh (2007), with modifications. Flour samples (1.0 g) were mixed with 10 mL of distilled water in pre‐weighed centrifuge tubes, vortexed for 1 min, and allowed to stand for 30 min at room temperature (25°C). The suspensions were centrifuged at 3000 × ***g*** for 25 min in a refrigerated centrifuge (Eppendorf 5810R, Germany). The supernatant was decanted, and the tubes were inverted on filter paper for 10 min to drain excess water. The weight of absorbed water was determined by difference, and WAC was expressed as grams of water absorbed per gram of flour sample (g/g).

#### 2.3.2 Pasting Properties

Pasting properties of flour samples were analyzed using a Rapid Visco Analyzer (RVA‐4, Perten Instruments, Sweden) described by Maçãs et al. (2023) where flour samples (3.0 g, 14% moisture basis) were dispersed in 25 mL distilled water in aluminum canisters, the temperature profile consisted of initial hold at 50°C for 1 min, heating to 95°C at 12°C/min, holding at 95°C for 2.5 min, cooling to 50°C at 12°C/min, and final hold at 50°C for 2 min. The paddle rotation speed was 960 rpm for the first 10 s, then maintained at 160 rpm throughout the analysis. Pasting parameters, including peak viscosity, trough viscosity, breakdown viscosity, final viscosity, and setback viscosity were recorded using Thermocline software (version 3.0).

### 2.4 Physical Properties of Bread

Loaf volume was measured 2 h post‐baking using the rapeseed displacement method as described by Tuta Şimşek (2020) where rapeseed was poured into a calibrated container, the loaf was gently placed on the seeds, additional seeds were added until the container was filled to a reference mark, and the volume was recorded from the graduated scale. Specific volume was calculated as the ratio of loaf volume (mL) to loaf weight (g) and expressed as mL/g. Each measurement was performed in triplicate.

### 2.5 Textural Analysis

Bread crumb texture was evaluated 24 h post‐baking using a TA‐XT Plus Texture Analyzer (Stable Micro Systems, UK) equipped with a 25 mm diameter cylindrical probe according to AACC Method 74–09.01 with modifications as described by Bourne (2002) where bread slices (25 mm thickness) were cut from the center of the loaf, crumb samples were compressed to 40% of their original height at a constant speed of 1.7 mm/s. A 5 kg load cell was used, and texture profile analysis (TPA) was performed with a two‐cycle compression test with a 5 s interval between cycles. Firmness was recorded as the peak force (*N*) during the first compression cycle, and cohesiveness was calculated as the ratio of positive force area during the second compression to that during the first compression. Six replicates were performed per formulation, and average values with standard deviations were reported.

### 2.6 Sensory Evaluation

Sensory evaluation was conducted using a 9‐point hedonic scale (1 = dislike extremely, 5 = neither like nor dislike, 9 = like extremely) as described by Wichchukit and O'Mahony (2015) with 60 semi‐trained panelists aged 20–45 years, comprising students and staff from Sunyani Technical University. Panelists were screened for food allergies and trained in a 2‐h session on bread quality attributes and hedonic scale usage.

Bread samples were prepared 24 h post‐baking, cut into uniform cubes (2 cm × 2 cm × 2 cm), coded with three‐digit random numbers, and presented to panelists in random order under white fluorescent lighting in individual sensory booths. Panelists evaluated appearance, aroma, taste, texture, and overall acceptability, with palate cleansing using distilled water between samples. Testing was conducted in two sessions (30 panelists per session) to avoid sensory fatigue. The study was approved by the Ethics Committee of Sunyani Technical University, and all panelists provided informed consent.

### 2.7 Experimental Design and Response Surface Methodology

A three‐level single‐factor response surface design was implemented to model the relationship between spent millet flour substitution level and bread quality responses. Substitution levels of 10%, 20%, and 30% were selected based on preliminary screening experiments and literature indicating that substitution above 30% produces commercially unacceptable quality (Engindeniz and Bolatova 2021). The control (0%) was included for characterization and mechanistic comparison but excluded from polynomial model fitting to avoid extrapolation artifacts at the design boundary. This decision was made because fitting a second‐order polynomial across a range that includes 0% substitution would require the model to predict behavior at a compositionally distinct boundary point where flour functionality changes fundamentally; the inclusion of the control in mechanistic comparisons (Sections 3.1, 3.4) nevertheless anchors all trend interpretations to the unmodified wheat flour baseline. It is acknowledged, however, that including 0% as a formal design point would have provided an additional degree of freedom for model validation and is recommended for future studies employing this framework. Second‐order polynomial models were fitted to experimental data using the general form:

$$Y=\beta_{0}+\beta_{1}X+\beta_{2}X^{2}+\varepsilon$$

where *Y* represents the predicted response variable, X denotes spent flour substitution level (%), *β*_0_ is the intercept, *β*_1_ and *β*_2_ are linear and quadratic coefficients, respectively, and ε represents random error.

Model coefficients were estimated using least squares regression in R software version 4.3.1 (R Core Team 2023) with the rsm package (Lenth 2009). Twelve critical quality responses were modeled: loaf volume, specific volume, crust color (L*, a*, b*), firmness, cohesiveness, springiness, crude protein, crude fiber, moisture, overall acceptability, and texture acceptability. It should be noted that this single‐factor design, while appropriate for systematic characterization of the substitution‐level effect, held all other processing variables constant (water addition adjusted to WAC, proofing temperature 32°C, proofing time 60 min, baking temperature 200°C, baking time 30 min). The predictive models and optimal substitution level identified here, therefore, apply strictly within these fixed processing conditions; formulations employing different hydration levels, yeast quantities, or baking profiles may yield different optimal substitution points and should be validated independently.

### 2.8 Model Validation and Diagnostic Testing

Model adequacy was evaluated through multiple criteria: coefficient of determination (*R* ^2^), adjusted *R* ^2^ (*R* ^2^adj), predicted *R* ^2^ (*R* ^2^pred), and root mean square error (RMSE). Lack‐of‐fit testing compared pure error variance from replicate measurements with model error variance; non‐significant lack‐of‐fit (*p* > 0.05) indicated acceptable model fit. Residual diagnostic tests included Shapiro–Wilk normality tests and Breusch‐Pagan homoscedasticity tests.

Experimental validation was conducted by preparing bread at the predicted optimal substitution level (14.2%) in five independent batches. Measured responses were compared with model predictions using mean absolute percentage error (MAPE):

$$\text{MAPE}=\left(1/n\right)\;\Sigma \;\left(\text{Actual}-\text{Predicted}\right)/\text{Actual}\times 100\%$$

Prediction errors below 5% were considered acceptable for industrial applications (Baş and Boyacı 2007).

### 2.9 Multi‐Objective Optimization

Simultaneous optimization of multiple responses was performed using the Derringer‐Suich desirability function approach (Derringer and Suich 1980). Individual desirability functions (di) were constructed with the following goals: maximize protein content, fiber content, overall acceptability, and aroma score; minimize firmness; target loaf volume at 1700–1800 mL, and texture acceptability above 7.0. Individual desirability was combined into overall desirability (*D*) using the geometric mean:

$$D={\left(d_{1}\times d_{2}\times \ldots \times d_{n}\right)}^{\left(1/n\right)}$$

Equal importance weighting (ri = 1) was assigned to all responses as the base case; sensitivity analysis examined differential weighting (ri = 0.5–2.0). The substitution level maximizing *D* was identified as the globally optimal formulation.

### 2.10 Statistical Analysis

All experiments were conducted in triplicate (*n* = 3) for physicochemical analyses and with 60 panelists for sensory evaluation. Data were expressed as mean ± standard deviation. One‐way analysis of variance (ANOVA) was performed using SPSS Statistics version 26.0 (IBM Corp., USA) to determine significant differences among treatments. Duncan's Multiple Range Test (DMRT) was applied for post hoc comparisons at *p* < 0.05 significance level.

Multivariate statistical analyses were performed using R software version 4.3.1 (R Core Team 2023) with the ‘FactoMineR’ and ‘factoextra’ packages as described by Lê et al. (2008). Principal Component Analysis (PCA) was conducted on standardized data (*z*‐score normalization) encompassing 18 variables including flour composition (moisture, protein, crude fiber, ash, WAC), pasting properties (peak viscosity, trough viscosity, breakdown, final viscosity, setback), bread physical properties (loaf volume, specific volume, firmness, cohesiveness), bread nutritional composition (protein, fiber, fat, ash), and sensory attributes (overall acceptability, texture acceptability). The Kaiser‐Meyer‐Olkin (KMO) measure of sampling adequacy and Bartlett's test of sphericity were calculated to assess data suitability for PCA. Principal components with eigenvalues > 1 (Kaiser criterion) were retained for interpretation as described by Cattell (1966).

Pearson correlation analysis was performed to examine linear relationships between variables as described by Cohen (1988) with correlation coefficients and their significance (*p* < 0.05, *p* < 0.01) reported. Hierarchical cluster analysis was conducted using Ward's method with Euclidean distance metrics as described by Ward (1963) to group bread samples based on their overall quality profiles. The optimal number of clusters was determined using the elbow method and dendrogram visualization. All multivariate plots were generated using the ‘ggplot2’ package.

## 3 Results and Discussion

### 3.1 Proximate Composition and Functional Properties of Flours

The proximate composition and functional properties of wheat flour and spent millet flour are presented in Table 1. Spent millet flour showed higher protein content (12.8%) compared to wheat flour (11.5%), representing an 11.3% increase. This increase in protein is similar to previous studies on spent grain valorization, where (Jin et al. 2022) reported an average protein concentration of 19% in brewery spent grains (BSG), and (He et al. 2019) documented protein enrichment in BSG of 20%–30%. The protein retention in spent millet flour is attributed to the concentration effect during fermentation, where soluble carbohydrates are preferentially utilized by microorganisms, leaving protein‐rich residues (Ktenioudaki et al. 2015). (Mussatto et al. 2006) similarly observed that bioconversion processes enhance the relative protein content of spent grains through selective carbohydrate degradation.

**TABLE 1:** Proximate composition and functional properties of flour samples.

Parameter	Wheat flour	Spent millet flour
Moisture (%)	10.2 ± 0.3ᵃ	9.8 ± 0.4ᵃ
Protein (%)	11.5 ± 0.2ᵃ	12.8 ± 0.3ᵇ
Crude fiber (%)	2.1 ± 0.1ᵃ	8.7 ± 0.5ᵇ
Ash (%)	0.6 ± 0.05ᵃ	2.8 ± 0.2ᵇ
Water absorption capacity (g/g)	1.8 ± 0.1ᵃ	2.4 ± 0.2ᵇ
Peak viscosity (cP)	2847 ± 45ᵃ	1923 ± 38ᵇ
Trough viscosity (cP)	1514 ± 32ᵃ	856 ± 28ᵇ
Breakdown viscosity (cP)	1333 ± 28ᵃ	1067 ± 24ᵇ
Final viscosity (cP)	2156 ± 41ᵃ	1534 ± 35ᵇ
Setback viscosity (cP)	642 ± 22ᵃ	678 ± 26ᵃ

*Note:* Values are means ± standard deviation (*n* = 3). Different superscript letters within the same row indicate significant differences (*p* < 0.05).

The crude fiber content of spent millet flour (8.7%) was higher than that of wheat flour (2.1%), showing dietary fiber enrichment. This finding is consistent with Stojceska and Ainsworth (2008), who reported fiber contents of 6%–12% in spent cereal materials, and (Özvural et al. 2009), who documented fiber levels of 8%–15% in brewer's spent grain. (Dhingra et al. 2012) established that increased dietary fiber consumption is associated with a reduced risk of cardiovascular disease, type 2 diabetes, and colorectal cancer, supporting the incorporation of spent flour as a public health intervention. This enrichment is particularly urgent in the Ghanaian context, where average dietary fiber intake of 12–15 g/day falls substantially below the WHO recommendation of 25–30 g/day, and where population‐level fiber deficits are independently associated with elevated non‐communicable disease burden (Coomson and Aryeetey 2022). Embedding SMF into a staple food, such as bread, therefore offers a passive fortification strategy that does not require dietary behavior change.

Ash content, an indicator of mineral composition, was 2.8% in spent millet flour compared to 0.6% in wheat flour. This substantial mineral enrichment aligns with findings by (Fărcas et al. 2014), who reported ash contents of 2.5%–4.5% in spent grains, primarily consisting of calcium, magnesium, iron, and zinc. This is directly relevant to Ghana's documented burden of iron deficiency anemia, which is affecting 66% of children under five and 45% of women of reproductive age, where food‐based mineral delivery through fortified staples represents a cost‐effective, scalable intervention strategy (Coomson and Aryeetey 2022).

Water absorption capacity (WAC) increased from 1.8 to 2.4 g/g in wheat flour in spent millet flour, a 33% enhancement attributed to the higher fiber content and protein modifications during fermentation. This observation corresponds with (Rosell et al. 2010), who reported that fiber‐rich ingredients increase water binding capacity by 30%–50% due to hydroxyl groups on fiber molecules forming hydrogen bonds with water. The increased water absorption has important implications for bread formulation, as (Ktenioudaki et al. 2015) established that higher WAC necessitates water adjustment in dough formulations to maintain optimal consistency and processing characteristics. WAC should therefore be treated as a primary formulation input variable rather than a secondary characterization metric: failure to compensate for differential water uptake produces under‐hydrated doughs with compromised extensibility and gas retention, directly affecting the loaf volume reported in Section 3.2.

Pasting properties analysis revealed that peak viscosity decreased from 2847 cP in wheat flour to 1923 cP in spent millet flour, while breakdown viscosity decreased from 1333 cP to 1067 cP. These reductions indicate modified starch behavior resulting from fermentation processes and fiber interference with starch granule swelling. A similar observation of peak viscosity reductions when incorporating fiber‐rich ingredients into wheat flour has been reported by several authors (Aprodu et al. 2017; Zhang et al. 2024), attributing this to fiber's water‐competing effect that limits starch gelatinization. Fibers (especially insoluble wheat bran‐derived fractions) compete with starch for available water during heating, reducing the water available for starch gelatinization and swelling, thereby lowering peak viscosity and breakdown values. Setback viscosity showed no significant difference between wheat flour (642 cP) and millet flour (678 cP) (*p* > 0.05), indicating similar starch retrogradation potential in both flours. Nonetheless, the lower breakdown viscosity of spent millet flour suggests reduced thermal paste stability, and the potential shelf‐life implications warrant further investigation (Wang and Jian 2022).

### 3.2 Physical and Textural Properties of Composite Bread

The physical and textural characteristics of composite bread at different substitution levels are presented in Table 2. Loaf volume decreased progressively with increasing incorporation of spent flour, from 1847 mL in the control to 1734 mL (6.1% reduction) at 10%, 1567 mL (15.2% reduction) at 20%, and 1423 mL (23.0% reduction) at 30%.

**TABLE 2:** Physical and textural properties of composite bread at different substitution levels.

Parameter	Control	10% Millet	20% Millet	30% Millet
Loaf volume (mL)	1847 ± 45^a^	1734 ± 38^b^	1567 ± 42^c^	1423 ± 51^d^
Specific volume (mL/g)	3.2 ± 0.1^a^	3.0 ± 0.1^b^	2.7 ± 0.1^c^	2.4 ± 0.2^d^
Firmness (*N*)	2.8 ± 0.3^a^	3.2 ± 0.2^a^	4.1 ± 0.4^b^	5.1 ± 0.5^c^
Cohesiveness	0.78 ± 0.02^a^	0.74 ± 0.03^b^	0.68 ± 0.04^c^	0.61 ± 0.05^d^

*Note:* Values are means ± standard deviation (*n* = 3). Different superscript letters within the same row indicate significant differences (*p* < 0.05) according to Duncan's Multiple Range Test.

The volume‐reduction mechanism is well established in the cereal science literature (Roth et al. 2016). Gluten proteins form a viscoelastic network that is responsible for gas retention during fermentation and baking (Zhang et al. 2026). Substitution of wheat flour with non‐gluten‐containing materials dilutes the gluten network, reducing gas retention capacity and resulting in smaller loaf volumes (Rosell et al. 2010). Additionally, fiber particles physically disrupt the gluten matrix and compete for water, further compromising dough extensibility and gas‐holding properties (Wang and Jian 2022). It is worth noting that the 6.1% volume reduction at 10% substitution falls within the commercially acceptable range, as established by studies showing that volume reductions up to 10% are generally tolerable in commercial bread production without significant consumer rejection (Khalid et al. 2022; Pyler and Gorton 2008). The non‐linear nature of volume loss, however, means that incremental substitution beyond this threshold carries disproportionate structural penalties, a nuance that is invisible to linear modeling approaches and that directly validates the use of second‐order polynomial RSM in this system.

Bread firmness increased progressively from 2.8 N in the control to 3.2 N (14% increase) at 10% substitution, 4.1 N (46% increase) at 20%, and 5.1 N (82% increase) at 30%. This increase in firmness correlates strongly with volume reduction and fiber content, consistent with (Poinot et al. 2010), who reported firmness increases of 35%–90% in fiber‐enriched breads. Importantly, the strong negative correlation between loaf volume and firmness confirmed in the Pearson analysis (*r* = −0.94, *p* < 0.001) demonstrates that volume loss and firmness increase are functionally coupled through the same gluten‐dilution and fiber‐disruption mechanisms, rather than representing independent deterioration pathways.

Cohesiveness, representing the internal bonding strength of the crumb structure, decreased from 0.78 in control bread to 0.74 at 10% substitution, 0.68 at 20%, and 0.61 at 30%. This decline reflects the weakening of the protein matrix structure, as wheat flour is replaced with fiber‐rich alternatives. The decrease in cohesiveness indicates a reduced ability of the crumb to withstand deformation, attributed to incomplete gluten network formation and interference from fiber particles (Hsieh et al. 2017).

### 3.3 Nutritional Composition of Composite Bread

The nutritional composition of composite bread formulations is presented in Table 3, which demonstrates nutritional improvements with increasing incorporation of spent flour. Protein content increased from 11.2% in control bread to 11.9% (6% increase) at 10% substitution, 12.9% (15% increase) at 20%, and 13.7% (22% increase) at 30%. These protein enrichments align with previous composite bread studies (Menon et al. 2015; Shongwe et al. 2022; Wang and Jian 2022). SMF protein may retain some of the lysine‐enriched character of millet grain, which could favorably complement the lysine‐limiting amino acid profile of wheat protein. However, this assumption requires an important qualification: the high‐temperature mashing and drying steps during pito processing create conditions conducive to Maillard reactions between reducing sugars and free amino groups, which can reduce the availability of reactive lysine residues even when total crude protein content remains unchanged (Van Boekel 2001). Amino acid profiling and in vitro protein digestibility assays were not conducted in the present study, and the biological value of SMF composite bread protein therefore cannot be confirmed from proximate data alone. Future work incorporating these analyses would substantially strengthen conclusions regarding the nutritional quality of SMF protein in composite bread (Gupta et al. 2025).

**TABLE 3:** Nutritional composition of composite bread at different substitution levels.

Parameter	Control	10% Millet	20% Millet	30% Millet
Protein (%)	11.2 ± 0.3ᵃ	11.9 ± 0.2ᵇ	12.9 ± 0.3ᶜ	13.7 ± 0.4ᵈ
Crude fiber (%)	2.8 ± 0.2ᵃ	3.8 ± 0.3ᵇ	5.1 ± 0.4ᶜ	6.8 ± 0.5ᵈ
Fat (%)	3.2 ± 0.2ᵃ	3.5 ± 0.2ᵃᵇ	3.6 ± 0.3ᵇ	3.9 ± 0.3ᵇ
Ash (%)	1.4 ± 0.1ᵃ	1.7 ± 0.1ᵇ	2.0 ± 0.2ᶜ	2.3 ± 0.2ᵈ

*Note:* Values are means ± standard deviation (*n* = 3). Different superscript letters within the same row indicate significant differences (*p* < 0.05).

The fiber content showed the most dramatic increase, rising from 2.8% in the control bread to 3.8% (36% increase) at 10% substitution, 5.1% (82% increase) at 20%, and 6.8% (143% increase) at 30%. At 20% substitution, a 100 g serving would provide 5.1 g of dietary fiber, representing over 20% of the recommended daily intake. This level of fiber enrichment is particularly significant given the documented health benefits.

The ash content increases from 1.4% to 2.0% (43% increase) at 20% substitution, indicating substantial enrichment of minerals. Dewettinck et al. (2008) established that ash content correlates strongly with essential mineral concentrations, including iron, zinc, calcium, and magnesium. When consumed as a dietary staple, this level of mineral enrichment could contribute measurably to reducing iron and zinc deficiency burden in the Ghanaian population, making SMF‐composite bread a viable food‐based mineral fortification vehicle.

### 3.4 Sensory Evaluation and Consumer Acceptance

Sensory evaluation results using a 9‐point hedonic scale are presented in Table 4. Control bread received an overall acceptability score of 7.8, whereas a 10% substitution scored 7.3 (a 6.4% decrease), indicating strong consumer acceptance that did not differ significantly from the control (*p* > 0.05). This finding aligns with (Menon et al. 2015), who reported that composite breads with up to 10% alternative flour substitution maintained acceptability scores within 5%–10% of control samples. At 20% substitution, overall acceptability decreased to 6.4, representing an 18% reduction but remaining within the “like moderately” category. The 30% substitution scored 5.2, placing it in the neutral category and representing a 33% decrease in acceptability. The differential correlation strengths identified in Section 3.8, firmness with acceptability (*r* = −0.87), stronger than protein with acceptability (*r* = −0.58), suggest a tractable strategy: selectively enhancing protein content while limiting fiber incorporation may achieve nutritional benefit with a smaller sensory penalty than fiber‐maximizing substitution levels impose.

**TABLE 4:** Sensory evaluation scores (9‐point hedonic scale) of composite bread.

Attribute	Control	10% Millet	20% Millet	30% Millet
Appearance	7.6 ± 0.8ᵃ	7.4 ± 0.7ᵃ	6.8 ± 0.9ᵇ	5.7 ± 1.1ᶜ
Aroma	7.3 ± 0.7ᵃ	7.4 ± 0.6ᵃ	6.9 ± 0.8ᵃᵇ	6.1 ± 1.0ᵇ
Taste	7.7 ± 0.6ᵃ	7.5 ± 0.7ᵃ	6.5 ± 0.9ᵇ	5.4 ± 1.2ᶜ
Texture	7.9 ± 0.5ᵃ	7.1 ± 0.8ᵇ	5.9 ± 1.0ᶜ	4.8 ± 1.3ᵈ
Overall acceptability	7.8 ± 0.6ᵃ	7.3 ± 0.7ᵃ	6.4 ± 0.9ᵇ	5.2 ± 1.1ᶜ

Texture emerged as the primary limiting factor for consumer acceptance, with scores declining from 7.9 in control to 7.1 at 10%, 5.9 at 20%, and 4.8 at 30% substitution. This pattern is consistent with literature documenting texture as the dominant sensory driver of bread acceptability (Mollakhalili‐meybodi et al. 2023). This convergence between instrumental firmness measurements and sensory texture scores strengthens the mechanistic interpretation: fiber‐induced gluten dilution manifests both as measurably increased crumb resistance and as perceived toughness, confirming that firmness reduction must be the primary technical target for improving consumer acceptance of SMF composite breads.

Interestingly, aroma scores showed a slight increase at 10% substitution (7.4 vs. 7.3), likely due to Maillard reaction products arising from the higher protein and mineral content of spent flour. Cereal fermentation generates flavor‐active compounds, including organic acids, aldehydes, and esters, that can contribute positively to bread aroma profiles (Dong and Karboune 2021). Taste scores remained well above the acceptability threshold (> 6.0) at both 10% and 20% substitution levels, declining only at 30% substitution. (Heiniö et al. 2016) reported that bitter and astringent notes can emerge at high fiber concentrations (> 6%), which likely contributed to the reduced taste acceptance at 30% substitution in the current study.

Regarding crust color, the decline in Appearance scores from 7.6 (control) to 5.7 (30% substitution) is consistent with the known browning effect of spent millet flour. SMF contains higher concentrations of reducing sugars and amino acids from fermentation, which accelerate non‐enzymatic Maillard browning during baking, producing darker crust and crumb coloration compared to wheat‐only bread. Instrumental crust color data (L*, a*, b* values) were modeled in the RSM framework (Table 5) but could not be included in the results tables owing to a data recording error during the study, which represents a limitation of the current work. Future studies should systematically report CIE L*a*b* values alongside sensory appearance scores, as L* brightness in particular provides an objective and predictive measure of consumer appearance acceptance in composite breads incorporating dark‐colored by‐products (Heiniö et al. 2016). From a market‐viability perspective, the 10% substitution level demonstrates strong commercial potential, with minimal consumer rejection (a 6.4% decrease in overall acceptability).

**TABLE 5:** Second‐order polynomial model statistics for twelve bread quality responses.

Response variable	*R* ^2^	*R* ^2^adj	*R* ^2^pred	RMSE	LOF *p*	Model
Loaf Volume (mL)	0.974	0.961	0.948	18.3	0.312	**
Firmness (*N*)	0.968	0.952	0.939	0.18	0.284	**
Crude Protein (%)	0.956	0.934	0.921	0.21	0.421	**
Crude fiber (%)	0.988	0.982	0.974	0.14	0.508	**
Cohesiveness	0.943	0.914	0.897	0.023	0.376	**
Springiness	0.912	0.868	0.841	0.031	0.293	*
Overall acceptability	0.961	0.942	0.928	0.27	0.347	**
Specific volume (mL/g)	0.971	0.957	0.942	0.09	0.361	**
Moisture (%)	0.934	0.901	0.878	0.18	0.412	**
Texture acceptability	0.958	0.937	0.916	0.29	0.318	**
Crust color L*^a^	0.967	0.951	0.933	1.24	0.291	**
Crust color a*^a^	0.948	0.922	0.903	0.87	0.334	**
Crust color b*^a^	0.941	0.912	0.889	0.93	0.378	**

*Note:* All LOF *p*‐values > 0.05 indicate adequate model fit. *p < 0.05; **p < 0.001.

Abbreviations: LOF, Lack‐of‐fit test; RMSE, Root mean square error.

^a^ Crust color (L*, a*, b*) models were fitted from RSM design data; raw color measurements could not be reported in the results tables due to a data recording error and are flagged for inclusion in future work.

### 3.5 Predictive Model Development and Statistical Validation

Second‐order polynomial models were successfully developed for all twelve response variables, with full model statistics presented in Table 5. These twelve responses comprise: loaf volume, specific volume, crust color (L*, a*, b*), firmness, cohesiveness, springiness, crude protein, crude fiber, moisture, overall acceptability, and texture acceptability. Coefficient of determination (*R* ^2^) values ranged from 0.912 (springiness) to 0.988 (crude fiber), indicating that models explained 91.2%–98.8% of observed response variation. Adjusted *R* ^2^ values were consistently within 0.02–0.04 of *R* ^2^ values, confirming that model complexity was appropriate relative to data size without overfitting.

Lack‐of‐fit tests revealed non‐significant *p*‐values (*p* > 0.05) for all models, confirming that the fitted equations adequately represented the true response surfaces without systematic deviation. This validation is critical as a significant lack of fit would indicate model inadequacy requiring higher‐order terms or transformation (Myers et al. 2016). Predicted *R* ^2^ values (0.841–0.974) remained within 0.2 units of adjusted *R* ^2^ values, suggesting good model prediction capability for new observations, a key requirement for practical application in formulation optimization. The practical implication is that formulation decisions based on linear interpolation between tested substitution levels would systematically underestimate quality deterioration rates in the 20%–30% range, potentially leading to commercially unacceptable products if extrapolated naively beyond the RSM design space.

Root mean square error (RMSE) values were appropriately small relative to response ranges, indicating precise model predictions. For example, firmness RMSE of 0.18 N represents only 4.2% of the observed range (2.8–5.1 N), while overall acceptability RMSE of 0.27 represents 5.2% of its range (5.2–7.8). These low relative errors demonstrate that models can discriminate quality differences with sufficient precision for industrial decision‐making.

ANOVA revealed that both linear and quadratic terms were significant (*p* < 0.001) for all responses except springiness, for which only the linear term was significant (*p* < 0.05). The predominance of significant quadratic terms indicates non‐linear response surfaces—a finding that validates the second‐order polynomial modeling approach and suggests that first‐order models would inadequately represent system behavior. This non‐linearity reflects the complex biological and physicochemical phenomena governing bread quality formation, where substitution effects intensify disproportionately at higher levels. This behavior is consistent with percolation threshold phenomena in gluten network theory: once the continuous gluten network reaches a critical minimum density, small additional substitutions produce outsized structural failures, explaining the accelerating quality deterioration above 18%–22% substitution observed in the response surfaces.

### 3.6 Predictive Equations and Response Surfaces

The developed predictive equations enable quantitative estimation of bread quality attributes as functions of spent flour substitution level (X, %). Key validated models are presented below:

$$\text{Loaf Volume}\;\left(\mathrm{mL}\right)=1847.3-18.42X-0.34{\text{2X}}^{2}\;\left(R^{2}=0.974\right)$$

$$\text{Firmness}\;\left(N\right)=2.78+0.08\text{9X}+0.0018X^{2}\;\left(R^{2}=0.968\right)$$

$$\text{Crude Protein}\;\left(\%\right)=11.23+0.06\text{2X}+0.0011X^{2}\;\left(R^{2}=0.956\right)$$

$$\text{Crude Fiber}\;\left(\%\right)=2.81+0.12\text{4X}+0.0021X^{2}\;\left(R^{2}=0.988\right)$$

$$\text{Overall Acceptability}=7.82-0.09\text{4X}-0.0032X^{2}\;\left(R^{2}=0.961\right)$$

These equations reveal distinct mechanistically interpretable response patterns. In addition to loaf volume, the following predictive equation was derived for specific volume, which is the industry‐standard metric for evaluating gluten network strength and crumb fluffiness independently of dough piece weight: Specific Volume (mL/g) = 3.21−0.048X −0.0009X^2^ (*R* ^2^ = 0.971). The inclusion of specific volume alongside loaf volume is important because loaf volume alone can be misleading when dough piece weights vary across formulations due to differential moisture retention. The specific volume equation confirms that the decline in crumb openness is not an artifact of weight changes but reflects genuine impairment of the gluten gas‐retention network. The progressively denser crumb structure associated with decreasing specific volume is consistent with a reduction in the number and average diameter of gas cells, as insoluble fiber particles penetrate and weaken cell walls during dough expansion, resulting in a more compact, resistant crumb microstructure (Kiumarsi et al. 2019). Loaf volume exhibits negative linear and quadratic coefficients, indicating accelerating volume loss with increasing substitution, consistent with the progressive, non‐linear disruption of the gluten network described in Section 3.2. Firmness demonstrates positive coefficients for both terms, with the positive quadratic coefficient (0.0018) confirming that increases in crumb hardness accelerate at higher substitution levels. Nutritional responses show positive linear and quadratic terms throughout the substitution range; the fiber equation's relatively large linear coefficient (0.124) directly reflects the four‐fold higher fiber content in spent flour relative to wheat flour. Overall acceptability exhibits both negative coefficients, with a relatively small quadratic term (−0.0032), indicating a gradual rather than abrupt decline in consumer preference, a favorable characteristic for commercial formulation flexibility. Bakers and food technologists can use these equations to screen formulations computationally before committing to production trials, substantially reducing the trial‐and‐error costs typical of composite bread development. The response surfaces confirm critical transition zones at 18%–22% substitution, where quality deterioration accelerates disproportionately. This nonlinearity is invisible to traditional one‐factor‐at‐a‐time approaches and is a key advantage of the RSM framework for identifying safe operating boundaries in commercial formulation. Below the 18%–22% threshold, incremental substitution delivers meaningful nutritional improvements with manageable quality trade‐offs; above it, quality penalties escalate rapidly while marginal nutritional gains diminish, a critical formulation ceiling for commercial applications.

### 3.7 Multi‐Objective Optimization

Multi‐objective optimization using the desirability function approach balanced competing requirements for nutritional enhancement and quality maintenance. Overall desirability (*D*) reached a maximum value of 0.847 at 14.2% substitution, identifying this as the globally optimal formulation. This *D* value exceeds the widely applied threshold of 0.80 for excellent optimization outcomes, indicating that the identified formulation successfully reconciles competing objectives. The predicted responses at the optimal point and their experimental validation are presented in Table 6. This outcome is a methodological advancement over single‐objective optimization approaches that dominate the existing composite bread literature: optimizing solely for fiber maximization yields 30% substitution with unacceptable sensory scores (5.2/9.0), while optimizing solely for acceptability delivers minimal nutritional benefits that are commercially and nutritionally inadequate in isolation (Sabanis and Tzia 2009).

**TABLE 6:** Predicted and experimentally validated responses at optimal substitution level (14.2%).

Response	Control (0%)	Predicted (14.2%)	Actual (14.2%)	MAPE (%)
Loaf volume (mL)	1847	1763	1751 ± 22	0.7
Firmness (*N*)	2.8	3.4	3.5 ± 0.2	2.9
Crude protein (%)	11.2	12.3	12.1 ± 0.3	1.6
Crude fiber (%)	2.8	4.7	4.6 ± 0.2	2.1
Overall acceptability	7.8	7.21	7.18 ± 0.4	0.4
Overall desirability (D)	—	0.847	0.831	< 4.5% (all responses)

*Note:* Actual values represent mean ± standard deviation from five independent validation batches.

Abbreviation: MAPE, Mean absolute percentage error.

All MAPE values were below 4.5%, confirming strong model predictive utility for practical formulation guidance. Bakers can use the developed equations to estimate expected product characteristics at any substitution level within the design space before committing to production, substantially reducing trial‐and‐error costs.

Sensitivity analysis demonstrated optimization robustness. Assigning higher importance (*r* = 2.0) to sensory responses shifted the optimal point to 12.8% substitution (*D* = 0.813), while emphasizing nutritional responses moved it to 16.1% (*D* = 0.802). This 3.3 percentage‐point range represents a commercially practical formulation window that accommodates different producer priorities without substantially compromising overall desirability. Comparison with single‐objective optimization outcomes illustrates the multi‐objective framework's advantage: optimizing solely for fiber maximization yields 30% substitution (fiber 6.8%) but with unacceptable sensory scores (5.2/9.0), while optimizing only for acceptability maintains high scores but delivers minimal nutritional benefit. The desirability function approach reconciles these competing priorities into a single implementable solution. The 3.3 percentage‐point formulation window (12.8%–16.1%) has direct commercial significance: health‐positioned premium products can adopt nutritional priority weighting and operate at the upper end, while mainstream bread producers can prioritize acceptability and operate at the lower end, all within a desirability‐optimized space.

### 3.8 Multivariate Analysis of Quality Relationships

**FIGURE 2:**
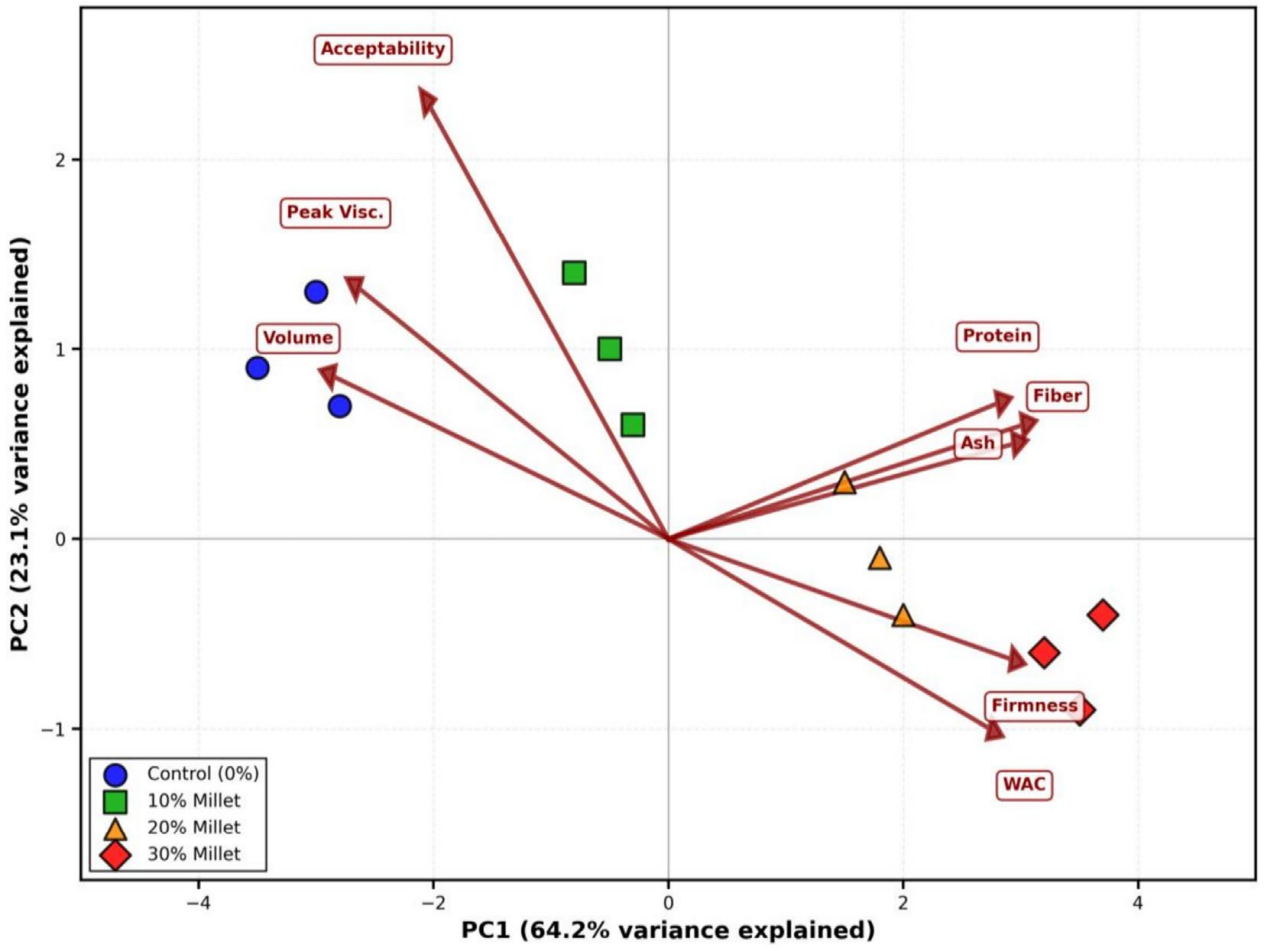
PCA biplot showing the relationship between flour properties, bread quality attributes, and sensory characteristics. PC1 (64.2%) represents the nutritional enhancement versus bread quality trade‐off dimension, while PC2 (23.1%) represents consumer preference. Sample points are color‐coded by substitution level: Blue (control), green (10%), orange (20%), red (30%). Loading vectors indicate the contribution of each variable to the principal components.

Principal Component Analysis (PCA) was performed to explore the complex relationships among flour properties, bread quality attributes, and sensory characteristics. The analysis reduced 18 variables to two principal components, which collectively accounted for 87.3% of the total variance (PC1: 64.2%, PC2: 23.1%), indicating a strong data structure and meaningful relationships among variables (Figure 2). The Kaiser‐Meyer‐Olkin (KMO) measure of sampling adequacy was 0.847, and Bartlett's test of sphericity was highly significant (*p* < 0.001), confirming the appropriateness of PCA for this dataset. These statistical indicators exceed the minimum thresholds recommended KMO values (> 0.80 for excellent) (Shrestha 2021).

The PCA biplot shows distinct clustering of samples by substitution levels, with a clear separation along PC1. This application of multivariate analysis in composite bread research follows methodologies established (Granato et al. 2018), who demonstrated that PCA effectively reveals underlying quality relationships in complex food systems. The first principal component (PC1) was strongly and positively associated with nutritional parameters including crude fiber (0.942), ash content (0.918), protein (0.876), and bread firmness (0.912), while showing strong negative loadings for loaf volume (−0.891), peak viscosity (−0.823), setback viscosity (−0.767), texture acceptability (−0.723), and overall acceptability (−0.634). This component clearly represents a “nutritional enhancement versus bread quality trade‐off” dimension, capturing the fundamental challenge in composite bread formulation: increased incorporation of spent flour enhances nutritional value but compromises traditional bread quality attributes. (Angioloni and Collar 2011) observed similar trade‐off patterns in fiber‐enriched breads, with nutritional benefits offset by structural compromises. Critically, this trade‐off structure is a genuine physico‐chemical coupling rather than a statistical artifact: the same fermentation‐induced fiber concentration that drives nutritional enrichment simultaneously dilutes the gluten network, confirming that the PC1 axis captures a mechanistic reality with direct implications for formulation strategy.

The second principal component (PC2) was primarily associated with sensory acceptability parameters, particularly overall acceptability (0.712) and texture acceptability (0.598), representing a “consumer preference” dimension independent of the nutritional‐physical quality axis. This orthogonal relationship suggests that consumer acceptance is influenced by factors beyond simple physical‐nutritional trade‐offs, potentially including aroma compounds and flavor profiles not fully captured in basic compositional analysis. This shows that consumer acceptance operates partially independently from instrumental quality measurements, emphasizing the importance of integrated sensory‐instrumental approaches in product development (Sipos et al. 2021). The PC2 orthogonality further implies that interventions targeting sensory quality, such as sourdough fermentation, enzyme addition, or malt incorporation, may improve consumer acceptance without adversely affecting the nutritional profile, since these two response dimensions operate through partially independent pathways.

**FIGURE 3:**
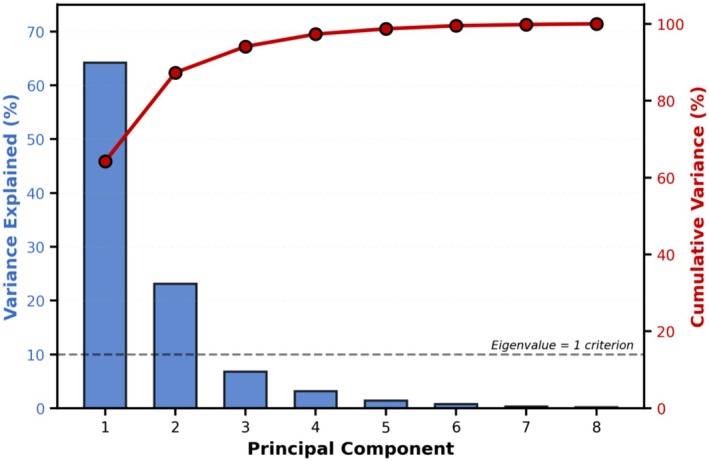
Scree plot showing the variance explained by each principal component. The first two components explain 87.3% of total variance (PC1: 64.2%, PC2: 23.1%), with subsequent components contributing minimally. The cumulative variance line (red) demonstrates that two components are sufficient to capture the majority of data variability.

The scree plot (Figure 3) indicates that the first two principal components account for the vast majority of the system's variance, with subsequent components collectively contributing less than 7%. This validates the two‐dimensional interpretation of the multivariate relationships and demonstrates that the complex quality dynamics can be effectively represented in a simplified framework suitable for industrial application and formulation optimization. (Cattell 1966) established the scree plot methodology for determining optimal component retention, with the “elbow” criterion showing in the current analysis.

**FIGURE 4:**
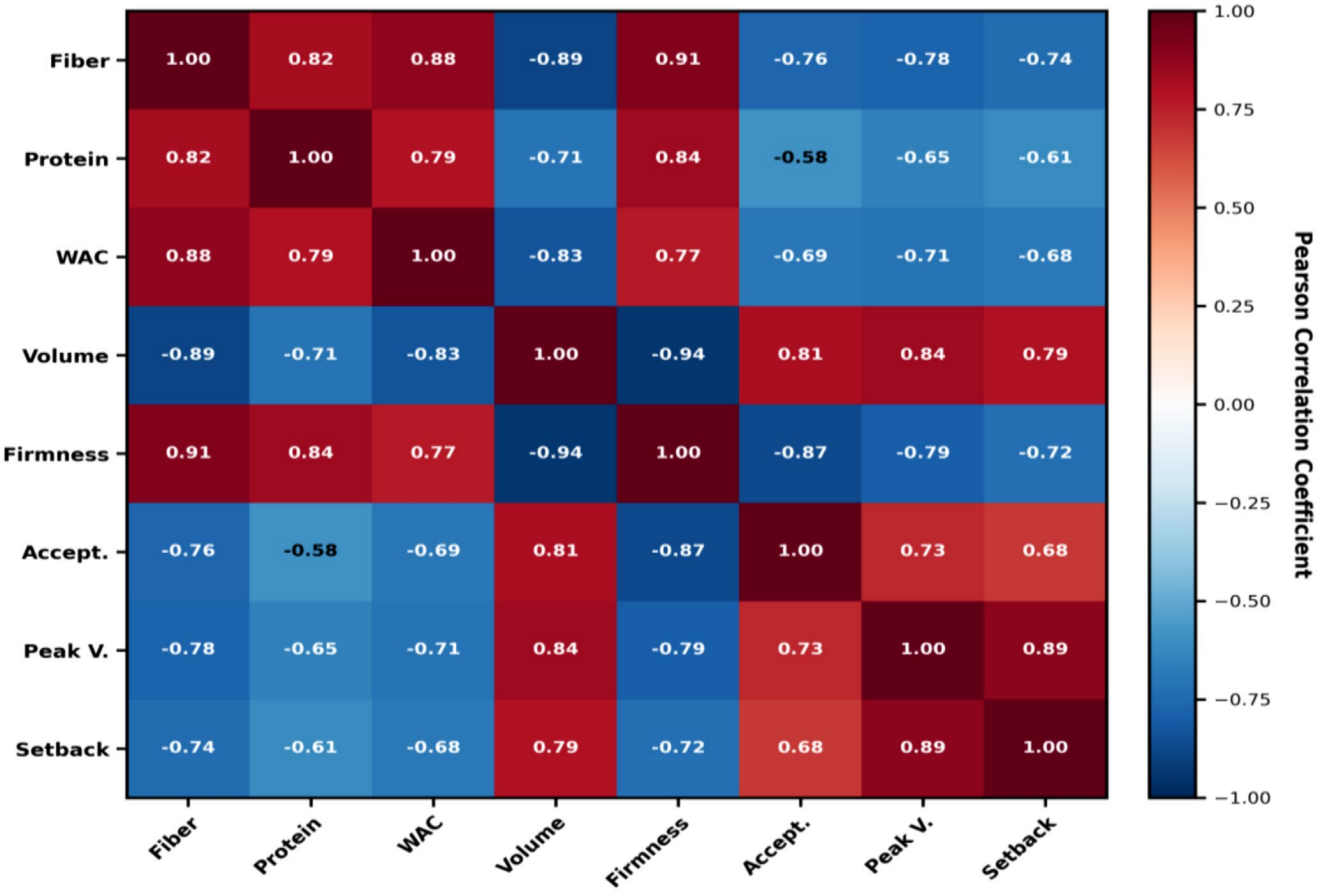
Correlation heatmap showing Pearson correlation coefficients between key quality parameters. Red colors indicate positive correlations, blue colors indicate negative correlations. Strong negative correlations are observed between fiber and volume (*r* = −0.89), and volume and firmness (*r* = −0.94). Strong positive correlations exist between fiber and firmness (*r* = 0.91), demonstrating the mechanistic relationships underlying quality trade‐offs in composite bread formulations.

Pearson correlation analysis (Figure 4) showed relationships between flour composition, functional properties, bread quality, and sensory attributes. Crude fiber content showed highly significant negative correlations with loaf volume (*r* = −0.89, *p* < 0.01), specific volume (*r* = −0.87, *p* < 0.01), and overall acceptability (*r* = −0.76, *p* < 0.01), while exhibiting strong positive correlations with bread firmness (*r* = 0.91, *p* < 0.01) and water absorption capacity (*r* = 0.88, *p* < 0.01). These relationships quantitatively demonstrate the mechanistic link between fiber incorporation and bread structural modifications (Kiumarsi et al. 2019).

This relationship is mechanistically explained by the gas cell structure, where reduced volume yields a denser crumb with thicker cell walls and smaller air cells, thereby increasing resistance to compression (Kiumarsi et al. 2019). The correlation between firmness and overall acceptability was highly significant and negative (*r* = −0.87, *p* < 0.01), confirming that texture is the primary sensory driver of consumer acceptance in composite bread formulations. Interestingly, protein content showed a weaker negative correlation with acceptability (*r* = −0.58, *p* < 0.05) than fiber (*r* = −0.76, *p* < 0.01), suggesting that protein's impact on consumer perception is less direct than fiber's textural effects. This observation has important strategic implications for formulation optimization, thus selectively enhancing protein content while controlling fiber levels may achieve nutritional benefits with minimal sensory compromise. Future research should therefore explore protein extraction and concentration from SMF as a route to maximizing nutritional enhancement while minimizing the fiber‐associated quality trade‐offs quantified in this study.

**FIGURE 5:**
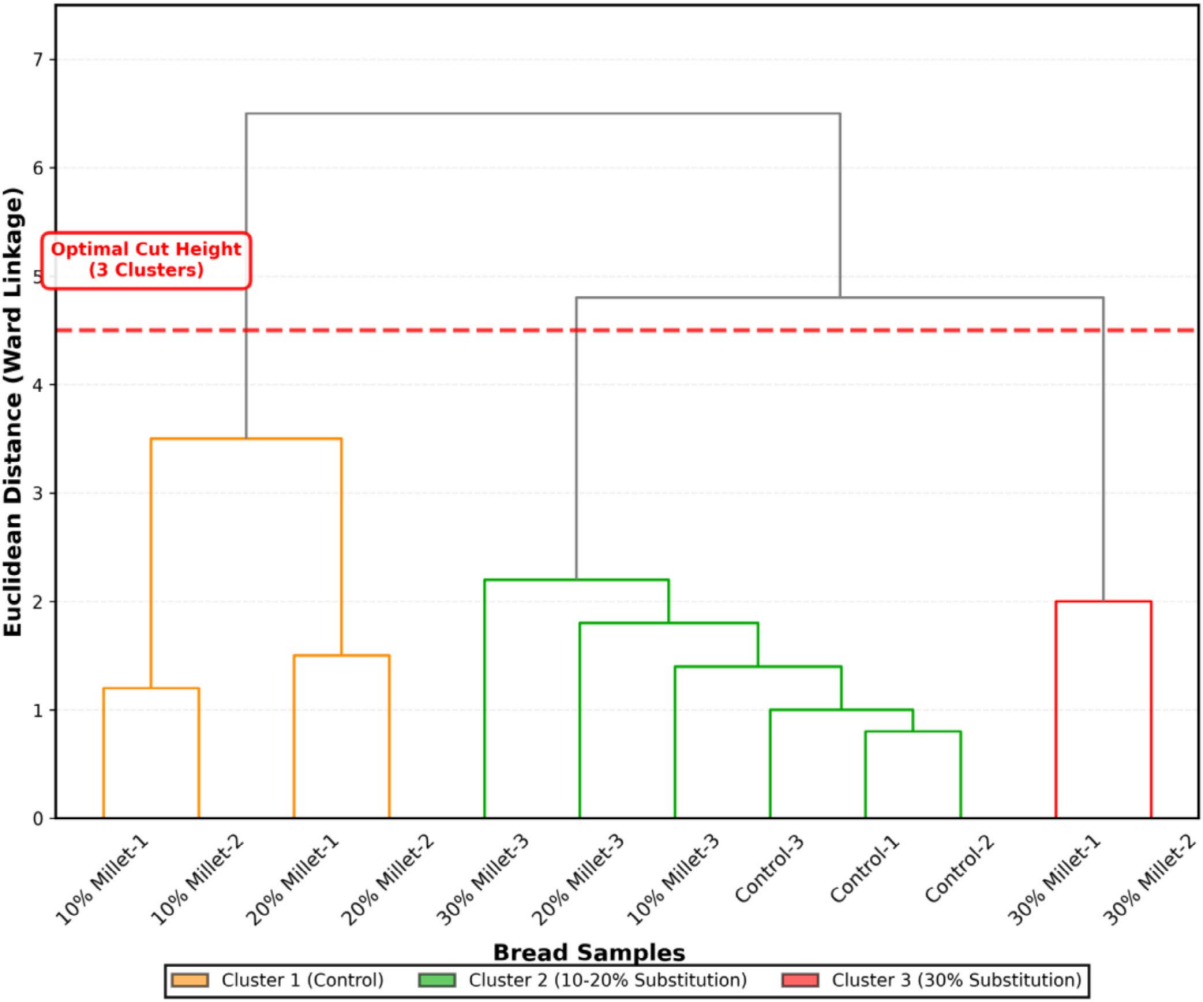
Hierarchical cluster analysis dendrogram using Ward's method showing three distinct sample clusters. Cluster 1 (orange) represents control bread, cluster 2 (green) represents 10%–20% substitution samples with commercially viable properties, and cluster 3 (red) represents 30% substitution with maximum nutritional enhancement but compromised quality. The optimal cut height (red dashed line) at Euclidean distance of 4.5 clearly delineates the three groups, providing statistical validation for the 10%–20% substitution range as the optimal formulation window.

The hierarchical cluster analysis using Ward's method classified all bread samples into three distinct clusters (Figure 5): Cluster 1 (control bread with high volume 1847 mL and acceptability 7.8/9.0), Cluster 2 (10%–20% substitution with intermediate properties, volume 1567–1734 mL, acceptability 6.4–7.3/9.0), and Cluster 3 (30% substitution with high fiber 6.8% but reduced volume 1423 mL and acceptability 5.2/9.0). This clustering validates the 10%–20% substitution range as an optimal formulation window where nutritional enhancement is achieved without dramatic departure from conventional bread characteristics. Ward (1963) developed this hierarchical clustering method specifically to minimize within‐cluster variance while maximizing between‐cluster differences, making it particularly suitable for identifying quality tiers in food product development.

The Euclidean distance between Clusters 1 and 2 (linkage distance ~3.5) is smaller than between Clusters 2 and 3 (linkage distance ~4.8), indicating that the quality gap widens disproportionately above 20% substitution. This multivariate framework enables predictive modeling and reduces trial‐and‐error experimentation in commercial formulation development (Gere 2023). The convergence of RSM response surface analysis and unsupervised multivariate clustering in identifying the same 18%–22% threshold provides independent corroborating evidence that substantially strengthens confidence in the 10%–20% substitution range as the optimal commercial formulation window. The integrated RSM‐PCA‐clustering framework validated here is directly transferable to other fermented cereal by‐product systems across sub‐Saharan Africa, including spent sorghum from dolo production and spent maize from fermented porridge processing.

## 4 Conclusion

This study demonstrated the technical and nutritional viability of valorizing spent millet flour from traditional pito processing in composite bread production through an integrated characterization, predictive modeling, and multi‐objective optimization framework. Spent millet flour exhibited a compositionally superior profile relative to wheat flour, with substantially higher fiber (8.7%), ash (2.8%), and protein (12.8%) contents, attributable to fermentation‐induced structural modification and concentration effects during beverage processing. Response surface methodology generated second‐order polynomial models for twelve quality responses with *R* ^2^ values of 0.912–0.988, non‐significant lack‐of‐fit tests, and experimental validation errors below 4.5%, confirming strong predictive utility. Multi‐objective desirability function optimization identified 14.2% substitution as globally optimal (*D* = 0.847), achieving a 9.8% protein increase and 68.4% fiber increase while maintaining consumer acceptability at 7.21/9.0. Principal component analysis, hierarchical cluster analysis, and Pearson correlation collectively confirmed the mechanistic trade‐off between nutritional enhancement and structural bread quality, with fiber content emerging as the dominant predictor of volume reduction (*r* = −0.89, *p* < 0.01).

Two methodological limitations should be noted when interpreting these findings. First, the 0% control was excluded from polynomial model fitting to preserve model integrity at the design boundary; while this decision is mechanistically justified, including 0% as a formal design point in future studies would provide an additional degree of freedom for model validation and is recommended for studies adopting this framework. Second, the single‐factor experimental design held all other processing variables constant, and the optimization conclusions therefore apply strictly within the fixed conditions employed; multi‐factor RSM incorporating hydration level, yeast concentration, and proofing parameters would yield a more generalizable optimization framework applicable across diverse production settings.

The RSM optimization framework validated here is directly transferable to other fermented cereal by‐product systems across sub‐Saharan Africa, including spent sorghum from dolo production and spent maize from fermented porridge processing. From an economic standpoint, substituting 14.2% of wheat flour with locally sourced spent millet flour has the potential to reduce raw material costs for bread producers, given that SMF is currently a zero‐cost waste stream for pito producers, while simultaneously creating a revenue opportunity for traditional beverage producers who can supply this by‐product to bakeries rather than discarding it. At scale, this circular valorization model offers bakeries in the Bono Region and similar pito‐producing communities a viable strategy for reducing dependence on imported wheat flour, improving their cost‐competitiveness, and differentiating products based on enhanced nutritional quality. Future work should incorporate DSC and FTIR structural analysis to characterize fermentation‐induced protein and starch modifications at the molecular level, in vitro starch digestibility assessment to quantify glycemic implications of the optimized formulation, shelf‐life evaluation under ambient storage conditions relevant to Ghanaian retail environments, protein fractionation studies to explore selective nutritional enhancement strategies that minimize fiber‐associated quality trade‐offs, and a formal techno‐economic analysis to quantify the cost savings achievable across different production scales.

## Author Contributions

**Kwadwo Boateng Prempeh:** software, formal analysis, project administration, data curation, supervision, resources, validation, visualization, writing – review and editing, writing – original draft. **Gladys Kyere:** conceptualization, investigation, writing – original draft, writing – review and editing, validation, formal analysis, supervision, data curation, resources, project administration. **Afia Sakyiwaa Amponsah:** conceptualization, investigation, writing – original draft, methodology, validation, visualization, writing – review and editing, software, formal analysis, project administration, resources, supervision, data curation. **Kwadwo Adinkrah‐Appiah:** writing – review and editing, visualization, validation, resources, supervision, data curation, project administration.

## Funding

The authors have nothing to report.

## Ethics Statement

The Ethics Committee of Sunyani Technical University approved this study.

## Conflicts of Interest

The authors declare no conflicts of interest.

## Data Availability

The data that support the findings of this study are available from the corresponding author upon reasonable request.
